# Urban flask measurements of CO_2_ff and CO to identify emission sources at different site types in Auckland, New Zealand

**DOI:** 10.1098/rsta.2022.0204

**Published:** 2023-11-27

**Authors:** Hayden A. Young, Jocelyn C. Turnbull, Elizabeth D. Keller, Lucas Gatti Domingues, Jeremy Parry-Thompson, Timothy W. Hilton, Gordon W. Brailsford, Sally Gray, Rowena C. Moss, Sara Mikaloff-Fletcher

**Affiliations:** ^1^ GNS Science, Lower Hutt 5010, New Zealand; ^2^ CIRES, University of Colorado at Boulder, Boulder, CO, USA; ^3^ Antarctic Research Centre, Victoria University of Wellington, Wellington, New Zealand; ^4^ Department of Atmospheric Sciences, Institute of Astronomy, Geophysics and Atmospheric Sciences, University of São Paulo, São Paulo, Brazil; ^5^ Greater Wellington Regional Council, Wellington, New Zealand; ^6^ National Institute of Water and Atmospheric Research (NIWA), Wellington, New Zealand

**Keywords:** carbon cycle, radiocarbon, urban emissions, fossil fuels, carbon dioxide, carbon monoxide

## Abstract

As part of the CarbonWatch-NZ research programme, air samples were collected at 28 sites around Auckland, New Zealand, to determine the atmospheric ratio (*R*_CO_) of excess (local enhancement over background) carbon monoxide to fossil CO_2_ (CO_2_ff). Sites were categorized into seven types (background, forest, industrial, suburban, urban, downwind and motorway) to observe *R*_CO_ around Auckland. Motorway flasks observed *R*_CO_ of 14 ± 1 ppb ppm^−1^ and were used to evaluate traffic *R*_CO_. The similarity between suburban (14 ± 1 ppb ppm^−1^) and traffic *R*_CO_ suggests that traffic dominates suburban CO_2_ff emissions during daytime hours, the period of flask collection. The lower urban *R*_CO_ (11 ± 1 ppb ppm^−1^) suggests that urban CO_2_ff emissions are comprised of more than just traffic, with contributions from residential, commercial and industrial sources, all with a lower *R*_CO_ than traffic. Finally, the downwind sites were believed to best represent *R*_CO_ for Auckland City overall (11 ± 1 ppb ppm^−1^). We demonstrate that the initial discrepancy between the downwind *R*_CO_ and Auckland's estimated daytime inventory *R*_CO_ (15 ppb ppm^−1^) can be attributed to an overestimation in inventory traffic CO emissions. After revision based on our observed motorway *R*_CO_, the revised inventory *R*_CO_ (12 ppb ppm^−1^) is consistent with our observations.

This article is part of the Theo Murphy meeting issue 'Radiocarbon in the Anthropocene'.

## 1. Introduction

CO_2_ emissions from fossil fuels (CO_2_ff) are the primary reason for the recent rapid increase in the atmospheric CO_2_ mole fraction [1]. Understanding these CO_2_ff emissions is crucial for assessing the global carbon budget and implementing the most effective emission reduction strategies. Urban areas account for just 3% of Earth's surface area yet produce approximately 70% of global fossil fuel emissions, the latter of which is expected to continue increasing [2]. For this reason, obtaining accurate emissions information has become essential for cities with goals of reducing their emissions.

Emissions information is well-established at the national and annual scale [3,4] but is less documented on the city scale. Recently, many cities have begun to inventory their emissions, often providing emission totals by sector [5–9]. The recent development of high-resolution emission maps for cities distribute CO_2_ff emissions spatially and temporally providing substantially more detailed information on urban emissions [4,10–17]. However, it can be difficult to quantify uncertainties and recent research has demonstrated that uncertainties are larger for individual source sectors than for the totals [4,18]. With goals of reducing greenhouse gas emissions by a few per cent per year, having emission information with uncertainties greater than reduction goals is undesirable [19].

Atmospheric measurements can substantiate and constrain bottom-up emissions information. Comparisons between inventory and atmospheric methods of emissions evaluation improves the accuracy of both methods. By combining these methods, a more effective quantification of CO_2_ff can be used to refine emission reduction strategies. Separating CO_2_ff from other CO_2_ sources and sinks is crucial for attribution and can be diagnosed from atmospheric measurements of the radiocarbon content of CO_2_ (^14^CO_2_), an excellent tracer for CO_2_ff [20–23].

During combustion, a small amount of carbon monoxide (CO) is produced by every CO_2_ff emission source. CO, while not a direct greenhouse gas, contributes to climate change through interactions with ozone, methane and CO_2_, and is a major air pollutant [24]. The magnitude of CO over background relative to CO_2_ff produced (*R*_CO_) varies greatly between CO_2_ff sources and depends on many factors such as the type of fuel used and the combustion efficiency of the process. In some cases, CO can be scrubbed using equipment such as a catalytic converter, which dramatically reduces the produced CO and observed *R*_CO_.

Since transport is a dominant emission source in most countries, transport *R*_CO_ has been the focus of many studies [25–30]. These studies have shown that transport *R*_CO_ varies regionally and temporally and primarily depends on the vehicle fleet composition, age and local emission control laws. New Zealand has few vehicle restrictions and as a result, would be expected to observe a greater CO output from transport when compared with emission-regulated cities like Paris and Zurich, and would be more similar to cities like Indianapolis, which has a similar level of emission regulation [29,31]. Average vehicle age also contributes to increased CO output, particularly in locations with few vehicle restrictions. Auckland, the city of focus, has a relatively high average vehicle age of 13 years, which is comparable with the US average of 12 years, but is significantly greater than France, Switzerland, the UK and many other countries [32–34]. Thus, Auckland measurements were expected to reflect a relatively high transport *R*_CO_. Inventory-based information and atmospheric observations show that residential, commercial and industrial emissions typically have a much lower *R*_CO_ than traffic [9,31,35], although this would be expected to range significantly depending on combustion conditions and emission controls [36,37].

In this study, we measure *R*_CO_, the ratio of excess (enhancement over background) carbon monoxide (COxs) to CO_2_ff, at different site types in Auckland, New Zealand, using atmospheric measurements. These *R*_CO_ measurements were then used to evaluate the relative contribution of traffic (with high *R*_CO_) to other Auckland CO_2_ff source sectors (with lower *R*_CO_).

## 2. Methods

### (a) Inventory data for CO_2_ff and CO

**Figure 1.**
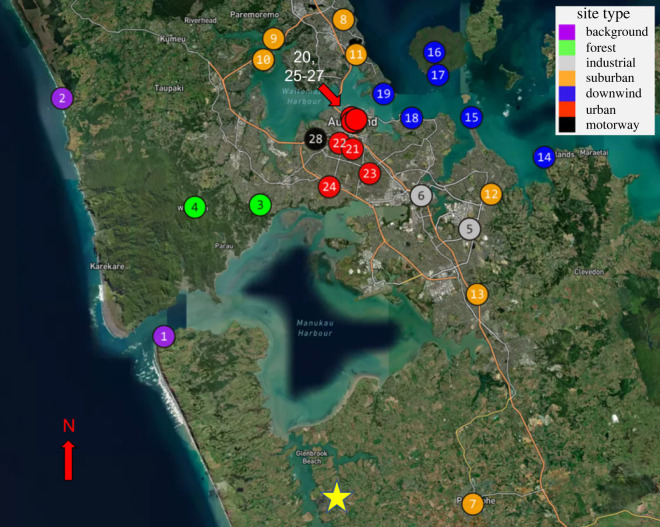
Map of Auckland with sites coloured by site type. Labels for each site correspond to the site IDs seen in table 1. The location of Glenbrook Steel Mill is marked with a star.

Auckland Council developed an air emissions inventory for the Auckland region in 2016 in accordance with the Global Protocol for Community-Scale Greenhouse Gas Emission Inventories [38–41]. In 2016, 65.8 kt of CO (66.8% transport, 28.1% domestic and 5.2% industrial) and 6852 kt of CO_2_ (59.1% transport, 6.6% domestic, 34.3% industry) was produced. From the inventory, we determine *R*_CO_ from each source sector, and for Auckland City as a whole, taking the ratio of CO:CO_2_ff from the inventory. It should be noted that Glenbrook Steel Mill (New Zealand Steel Limited) accounted for approximately 25% of Auckland's total CO_2_ff emissions and approximately 76% of Auckland's industrial CO_2_ff on its own in 2016 [38]. While Glenbrook is within the Auckland City jurisdiction, it is outside of the urban area (figure 1). Thus, we remove the emissions of both CO and CO_2_ff from Glenbrook from the inventory when calculating the source sector and whole city *R*_CO_ values.

**Table 1.** Sites observed around Auckland using flask data. Each site was assigned a site type depending on its location. The ID column corresponds to the site IDs in figure 1. Downwind sites that were situationally used as background under easterly winds are marked by an asterisk.

site name	type	altitude (m)	latitude	longitude	ID
Manukau Heads Lighthouse	background	234	−37.0507	174.5448	1
Muriwai	background	26	−36.8305	174.4268	2
Titirangi Woodfern Crescent Park	forest	59	−36.9293	174.6564	3
Waiatarua Community Centre	forest	253	−36.9314	174.5806	4
East Tāmaki	industrial	19	−36.9516	174.8990	5
Mount Wellington	industrial	11	−36.9207	174.8429	6
Pukekohe Bledisloe Park	suburban	64	−37.2055	174.9028	7
Forest Hill Greville Reserve^*^	suburban	18	−36.7575	174.7528	8
Greenhithe Collins Park	suburban	30	−36.7752	174.6719	9
Hobsonville Point Kōtuku Park	suburban	19	−36.7947	174.6600	10
Takapuna Auburn Reserve	suburban	18	−36.7899	174.7676	11
Botany Downs Our Lady School	suburban	40	−36.9193	174.9240	12
Manurewa Auckland Botanical Garden	suburban	61	−37.0117	174.9078	13
Beachlands Hawkes Crescent^*^	downwind	5	−36.8851	174.9856	14
Musick Point^*^	downwind	13	−36.8482	174.9012	15
Rangitoto Summit^*^	downwind	244	−36.7878	174.8580	16
Rangitoto Wharf	downwind	1	−36.8090	174.8624	17
Takaparawhā	downwind	52	−36.8488	174.8319	18
Takarunga (Mount Victoria)	downwind	84	−36.8264	174.7994	19
Sky Tower Level 61	urban	220	−36.8485	174.7621	20
Maungawhau (Mount Eden)	urban	193	−36.8775	174.7633	21
Kōwhai School	urban	19	−36.8721	174.7483	22
Maungakiekie (One Tree Hill)	urban	184	−36.9002	174.7832	23
Puketāpapa (Mount Roskill)	urban	194	−36.9125	174.7364	24
AUT WO building	urban	44	−36.8542	174.7655	25
Aotea Square	urban	26	−36.8523	174.7630	26
Albert Park	urban	52	−36.8501	174.7677	27
Western Springs Garden Community Hall	motorway	19	−36.8682	174.7205	28

### (b) Sampling site selection

Auckland is the largest city in New Zealand with a population of about 1.4 million people [42] and produces over 25% of New Zealand's CO_2_ff emissions [9,43]. Twenty-eight different sites were selected around the Auckland region. These sites observed a large variety of different emission types and were chosen to cover a large geographical area. The locations were classified into one of the following site types: background, forest, industrial, motorway, urban, suburban or downwind (figure 1 and table 1). Background sites were located on Auckland's coasts to measure incoming air masses (electronic supplementary material, figures S.1 and S.2). Under the prevailing south-westerly winds, these sites were Manukau Heads Lighthouse (MKH) and Muriwai (MW) on Auckland's West Coast, but under north-easterly wind, the downwind sites on Auckland's East Coast were categorized as background. The two forest sites were in or near to the Waitākere Ranges, a large, lightly populated forested region within the Auckland City boundary (electronic supplementary material, figures S.3 and S.4). The two industrial sites were in light industrial areas that contained machinery, chemical pollutants and heavy vehicles as well as traffic (electronic supplementary material, figures S.5 and S.6). The motorway site was close to the Auckland Northwestern Motorway (10 m away) and was expected to be strongly dominated by local traffic emissions (electronic supplementary material, figure S.28). Urban locations were identified by their higher population densities and were situated near to the Auckland Central Business District (CBD) (electronic supplementary material, figures S.20–S.27). Suburban locations were located outside of the inner city in Auckland's suburbs which are dominated by low-density, single-family housing (electronic supplementary material, figures S.7–S.13). Most of the sites fell into the urban and suburban site categories. Finally, the downwind sites were typically at elevated positions such that they observed emissions integrated over a larger area of Auckland, representing a more generalized Auckland emission signal (electronic supplementary material, figures S.14–S.19).

### (c) Sample collection

Whole air samples were collected 10 m above ground level by attaching an inlet tube to a telescopic mast to avoid sampling of immediate ground emissions. It should be noted that downwind sites were typically located at higher altitudes such as Auckland's volcanic cones or clifftops (table 1 and figure 1; electronic supplementary material, figures S.14–S.19), adding additional effective height. Each flask sample was captured using a sampler (Masker), which pumped ambient air through the inlet tube, a filter, a magnesium perchlorate trap (to remove particulates and moisture), through two pumps in parallel into an evacuated 2.5 l flask. The flasks were flushed with ambient air for 10 min at a flow rate of approximately 2.5 l min^−1^ before being pressurized to approximately 1 bar above atmospheric pressure over the course of approximately 1 min. Very local, short-term emission sources such as passing heavy traffic were avoided.

Field campaigns to collect flasks in Auckland were made every few months between October 2017 and February 2021. Sixteen campaigns were undertaken with 426 flasks being collected in total (electronic supplementary material, tables S.1, S.2 and S2.1). Air samples were collected primarily between 8.00 and 18.00. Since samples were collected manually, time of collection varied for each sample. In most cases, we collected the background samples earlier in the day than the other sites. A small set of samples was collected during COVID-19 lockdowns in April 2020 and these campaigns (two) were excluded from the dataset due to large changes in the observable emissions over the lockdown period [44].

CO and CO_2_ mole fractions were measured using cavity ring-down spectroscopy at NIWA using a Picarro G2401 [45] with precisions of 5 ppb and 0.05 ppm, respectively. Flow was restricted using a critical orifice to conserve sample air for subsequent analyses. To analyse ^14^CO_2_ in each sample, CO_2_ was isolated from each sample using cryogenic extraction [46], graphitized [47] and analysed in the Extended Compact Accelerator Mass Spectrometry system in the Rafter Radiocarbon Laboratory at GNS Science [48]. Results are reported as Δ^14^C [49].

### (d) Calculation of enhancements in CO and CO_2_ff

The ‘excess’ or enhancement in CO and CO_2_ff was determined for each sample, which was the added mole fraction as the air passed across Auckland City. First, the background incoming air CO mole fraction and Δ^14^C were determined. In most campaigns, when the wind direction was from the west or southwest, the two primary background sites (MKH and MW) were selected. Two flasks were collected for each campaign at each background site, typically collecting from one site on each day of the 2-day campaign. If all four samples showed consistent values for CO, CO_2_ and Δ^14^C, a weighted mean of all background flasks was used as a background for that campaign. If the two different days of background measurements showed significant differences in their reported compositions, each day was treated separately, averaging the two available background measurements for that day. The average representation error for the background flasks for CO, CO_2_ and Δ^14^C was 3 ppb, 1 ppm and 1‰, respectively, across all flask campaigns. This was calculated from the difference between the background values and the averaged background for each campaign. When the wind was from the east or northeast (Campaign 6, 8, 10 and 16, electronic supplementary material, table S.2), we instead used Beachlands Hawkes Crescent, Musick Point and Rangitoto Summit as background, following the same methodology of averaging across multiple measurements. MKH and MW were treated as downwind sites for those campaigns. For one campaign, the wind direction changed from southwest in the morning to northeast in the afternoon. Since most samples were collected in the afternoon on that day, Beachlands Hawkes Crescent and Musick Point were used as the background sites for both days of the campaign. MKH and MW, the two usual background sites, showed significantly greater CO over these 2 days and were classified as downwind sites. The chosen background sites and samples are listed in the electronic supplementary material for each campaign (electronic supplementary material, table S.2). The uncertainty in Δ^14^C for the background was derived by combining the measurement uncertainty of the background measurements with the uncertainty from the spread in Δ^14^C values measured at each background site [47].

CO_2_ff was calculated from the observed Δ^14^C (Δ_obs_), CO_2_ mole fraction (CO_2_obs) and Δ^14^C measured at the background site (Δ_bg_) (equation 2.1) [21,50],

2.1 $$\text{C}{\text{O}}_{2}\text{ff}=\;\frac{\text{C}{\text{O}}_{2}\text{obs}(\Delta_{\text{obs}}-\;\Delta_{\text{bg}})}{\Delta_{\text{ff}}-\;\Delta_{\text{bg}}}-\;\frac{\text{C}{\text{O}}_{2}\text{other}(\Delta_{\text{other}}-\;\Delta_{\text{bg}})}{\Delta_{\text{ff}}-\;\Delta_{\text{bg}}},$$

where Δ_ff_ is the Δ^14^C value for fossil fuel CO_2_ (−1000‰ by definition). The second term in the equation is a small bias term that adjusts CO_2_ff to allow for contributions of ^14^C from other sources such as heterotrophic respiration, ocean exchange and nuclear ^14^C sources. Since New Zealand has no nuclear sources and we sample the incoming air arriving on the western coast of Auckland, we assume that ocean and nuclear sources are included in the background measurement. Therefore, only heterotrophic respiration occurring between background and observing sites is corrected for. We use a bias value of −0.5 ± 0.2 ppm [22,51], and this value has been independently estimated for New Zealand from ^14^C in recent grass samples [44].

COxs was determined by subtracting the measured background CO mole fraction (CO_bg_) for a campaign from the observed CO mole fraction (CO_obs_) for each sample (equation 2.2).

2.2 $$\text{COxs}=\text{C}{\text{O}}_{\text{obs}}-\text{C}{\text{O}}_{\text{bg}}.$$

### (e) Determination of *R*_CO_ ratios

Plotting COxs against CO_2_ff produces a scatter plot with a gradient equal to the emission ratio *R*_CO_ [37,51–53]. To calculate the gradient, a York fit was chosen over an ordinary least squares (OLS) fit to calculate the line of best fit [54,55]. A York fit takes the uncertainty of both the dependent and independent variables into account and was used as the primary technique for calculating *R*_CO_ from the flasks [37,53,56–58]. The BFSL (best fit straight line) R package was used to create these York fits and determine the emission ratio and uncertainty [59].

Several outlier samples with higher/lower COxs than other samples from the same site during the 5-year measurement period showed unusually high or low *R*_CO_ values (determined from the observed COxs value, electronic supplementary material, table S.3). If the COxs of a sample was significantly greater/less (by more than 50 ppb, or 30 ppb for the forest sites due to reduced emissions at those locations) than the initial trend line fit for that site type, it was likely biased by a local emission source not representative of the site type, such as a car without a working catalytic converter or a nearby gas barbecue. These points were marked as outliers and a new trendline was fitted excluding these points. COxs was used instead of *R*_CO_ to determine outliers since *R*_CO_ tended to approach very high values when CO_2_ff approaches zero, despite being within the uncertainty of the trend line. Since each air sample was collected over approximately 1 min, an intermittent local emission source could dominate the measurement and increase the emission ratio observed from that measurement. Only a few outliers were removed from each site type dataset so *R*_CO_ did not change substantially, but the measured *r*^2^ values slightly increased. Additionally, points with much larger COxs and CO_2_ff than other samples could heavily constrain the *r*^2^ value determined from the correlation. These constraining points tended to increase *r*^2^ significantly such that they were less representative of the overall correlation of the dataset but changed *R*_CO_ minimally (within the *R*_CO_ uncertainty). If the constraining point(s) in the plot altered the York fit correlation substantially, they were also filtered from the dataset. After removing outliers, some constraining points and data collected over the COVID-19 lockdown period in New Zealand, the total number of samples examined was 346 (electronic supplementary material, table S.1). Each point that was filtered from the dataset is described in the electronic supplementary material, table S.3. *R*_CO_ was determined for each of the site types excluding the background site (motorway, urban, suburban, downwind, industrial and forest).

Small temporal changes to *R*_CO_ would be expected but observing annual changes was difficult with the limited data. Since 2018 and 2019 were the only measurement years with a full year worth of data, the flask data from each year were combined into a single dataset.

## 3. Results

### (a) CO and CO_2_ff magnitudes by site

**Figure 2.**
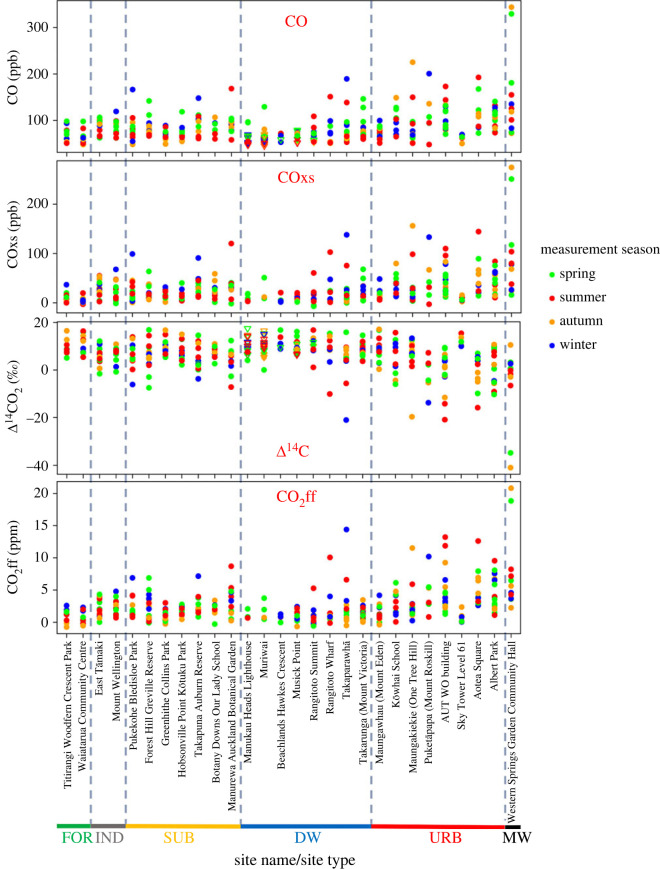
CO, COxs, Δ^14^C and CO_2_ff observed for each of the site measurements. Sites are ordered by site name and site type (left to right on the *x*-axis: forest, industrial, suburban, downwind, urban and motorway). Depending on the wind direction during each measurement day, different sites were used as background. These background measurements are marked as triangles in the CO and Δ^14^C graphs but were omitted from the COxs and CO_2_ff graphs. The measurement season of each sample is indicated by colour.

At the 28 sites, CO and Δ^14^C ranged from 45 to 344 ppb and from −41‰ to 18‰, respectively (figure 2). Overall, the motorway site had the greatest CO, lowest Δ^14^C and greatest range of values. High CO and low Δ^14^C were also observed at the urban, downwind and suburban locations. As expected, the smallest measured CO and the greatest measured Δ^14^C were at the background and forest sites with relatively low CO and high Δ^14^C also seen at the industrial sites.

COxs (equation 2.2) ranged from −7 to 275 ppb (figure 2). Since the background sites tended to have the lowest CO values due to few local emission sources, most excess values were positive, but a few measurements showed weakly negative values indicative of the uncertainty in the measurements and the choice of background.

Similarly, Δ^14^C was used to calculate CO_2_ff, which ranged from −1 to 21 ppm (figure 2). Samples with high CO_2_ff tended to also have high COxs.

The location and emission sources surrounding each site will influence the observed COxs and CO_2_ff values. This includes the source type, the magnitude of emissions from that source and the location of the source relative to the measurement site. Additionally, atmospheric transport has a significant influence on observed mole fractions [60]. Atmospheric transport disperses emissions so that sources closer to the measurement site have a larger impact on the observed mole fractions. High wind speeds and less stable air disperse emissions at a faster rate. While COxs and CO_2_ff were expected to increase in the winter due to an increase in residential and commercial emissions from heating [40], a seasonal change is not obvious in our mole fraction dataset (figure 2). Likely, competing influences from variable atmospheric transport are dominating seasonal changes in the variability in observed CO_2_ff and COxs.

### (b) Diagnosing *R*_CO_ for each emission sector

#### (i) Motorway site

**Figure 3.**
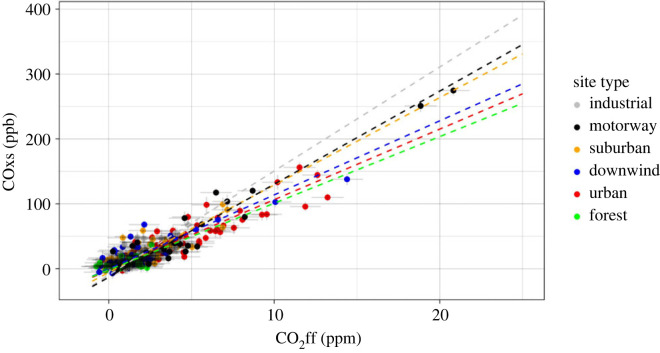
*R*_CO_ plot for each of the six site types.

The motorway site (Western Springs Garden Community Hall) had a relatively high *R*_CO_ of 14 ± 1 ppb ppm^−1^ and the greatest range in COxs and CO_2_ff (maximum COxs of 275 ppb and CO_2_ff of 21 ppm) (figure 3 and electronic supplementary material, figure S.34, table 2). The proximity of the site to the Auckland Northwestern Motorway and the high correlation coefficient (*r*^2^ = 0.9) of the plot indicated that the emission ratio measured at the motorway site was representative of Auckland's traffic emission ratio. While the assumption was made that the motorway site was dominated by traffic emissions, these measurements were also expected to observe small amounts of CO_2_ff from non-traffic sources. Unexpected non-traffic emissions would impact the validity of our results by biasing the traffic *R*_CO_ and our estimation of the traffic contribution to Auckland's emissions. However, due to the traffic density at the site, the motorway *R*_CO_ was expected to provide a good representation of Auckland's traffic *R*_CO_. Auckland's motorway *R*_CO_ also showed a strong similarity to the traffic *R*_CO_ measured for the US car fleet (approx. 15 ppb ppm^−1^), which was expected to be comparable with the Auckland car fleet due to similar emission regulations [26,27,29,31]. It should be noted that transport *R*_CO_ tends to decrease over time as newer cars replace older cars on the road [26,61]. As a result, older studies were expected to overestimate the current *R*_CO_ which introduces an uncertainty to this comparison.

**Table 2.** *R*_CO_ and *r*^2^ values measured for each of the six site types.

site type	*R*_CO_ (ppb ppm^−1^)	*r*^2^
motorway	14 ± 1	0.9
industrial	16 ± 3	0.4
suburban	14 ± 1	0.6
downwind	11 ± 1	0.8
urban	11 ± 1	0.8
forest	10 ± 4	0

One caveat is that different vehicle types and speeds can alter the observed *R*_CO_ [27,62–64]. *R*_CO_ is typically lower under motorway conditions (uninterrupted driving, warm engines) than in urban/suburban conditions (stop-and-go traffic, cold starts). Since our motorway site, Western Springs Garden Community Hall, is located between a motorway and a busy road, it represents a mixture of free-flowing and congested traffic. The measurement uncertainty of the observed *R*_CO_ was estimated from the York fit, but it remains difficult to estimate the uncertainty in traffic *R*_CO_ due to the different vehicle fleets/driving conditions at the other site types. Nonetheless, we expect that such bias in our traffic *R*_CO_ value would bias our *R*_CO_ value low.

**Figure 4.**
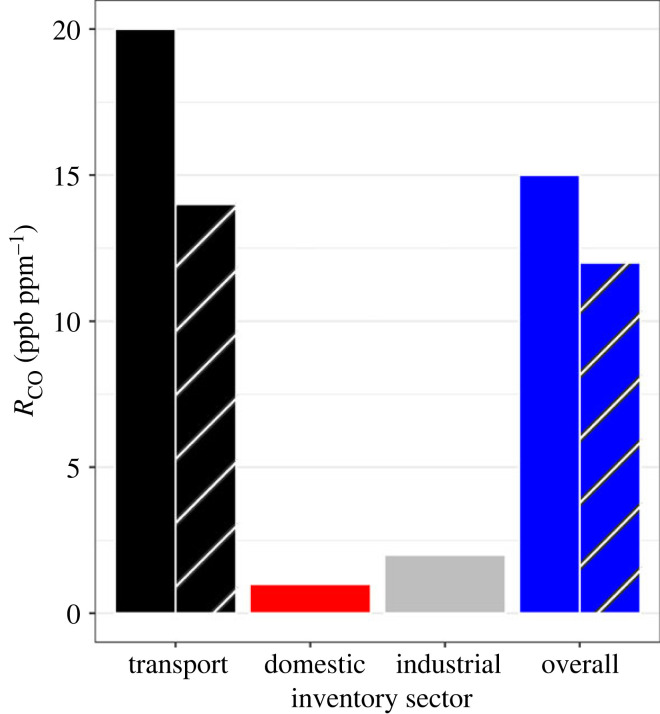
*R*_CO_ calculated from Auckland inventory for the transport, domestic and industrial sectors. Includes original inventory calculations (removing sources not expected to be observed during sample collection, solid bars) and revised inventory values (striped bars).

The 2016 inventory for Auckland estimates *R*_CO_ as 20 ppb ppm^−1^ for Auckland's traffic sector [40] (figure 4). There are several possible reasons for the discrepancy between the inventory and observed atmospheric value of 14 ± 1 ppb ppm^−1^. Inventory CO is determined by multiplying activity data by an emission factor assigned to each emission source. The uncertainty in the CO emission factor is stated to be 40% for petrol vehicles in addition to an approximate uncertainty of 22% for activity data [65]. Inventory CO has also been shown to be too high in a number of international studies so a similar overestimation is plausible for Auckland [22,31,37,51,52,66,67]. It is also possible that traffic CO_2_ff is underestimated by the inventory resulting in an *R*_CO_ that is too high. This seems unlikely since CO_2_ff is used to derive CO emissions in the inventory calculation [40]. Further, our observed *R*_CO_ is consistent with overseas studies of similar vehicle fleets and similar emission regulations that observed traffic *R*_CO_ values of approximately 15 ppb ppm^−1^ [26,27,29,31].

#### (ii) Urban, suburban and downwind sites

*R*_CO_ was similarly calculated for each sector from inventory data. Auckland's domestic inventory (residential and commercial emissions) includes emissions from coal (*R*_CO_ = 56 ppb ppm^−1^), LPG (*R*_CO_ = 0.1 ppb ppm^−1^), natural gas (*R*_CO_ = 0.4 ppb ppm^−1^), lawn mowers (*R*_CO_ = 390 ppb ppm^−1^) and wood/outdoor burning (CO and CO_2_bio produced, no CO_2_ff), which gives a total domestic *R*_CO_ of 166 ppb ppm^−1^ (table 3) [40].

**Table 3.** Table of domestic sector emissions [40]. Wood and outdoor burning are excluded since they do not typically occur during the time periods of our sampling campaigns.

domestic source	CO (t yr^−1^)	CO_2_ff (kt yr^−1^)	*R*_CO_ (ppb ppm^−1^)
coal	71	2	56
LPG	3	44	0.1
natural gas	29	122	0.4
wood burning	15 752	0	—
outdoor burning	877	0	—
lawn mowers	1726	7	387
total	18 458	175	166
total (coal, LPG, natural gas)	103	168	1

Since the flask samples collected in Auckland were collected between 8.00 and 18.00 on weekdays, we excluded wood burning from our estimates. While wood burning contributes about 99% of Auckland's household CO, minimal wood burning is expected for home heating during the sample collection period [40,68]. Wood burning is also minimal outside of the winter months (June, July, August), which was when over 75% of the samples were collected [68]. Additionally, outdoor burning, which is banned in Auckland's urban areas (allowed in rural areas within city boundary but would not be observed by our city measurements) and lawn mowing, which would be expected to be uncommon during the daytime on weekdays, were also omitted. Excluding these emissions leaves domestic emissions from coal, LPG and natural gas, which gives an inventory domestic *R*_CO_ of 1 ppb ppm^−1^ (figure 4) [40]. Auckland's industrial inventory includes emissions from steel production, glass making, chemical manufacturing and a number of other industrial processes [38]. Auckland's industrial source sector *R*_CO_ was calculated to be 1.6 ppb ppm^−1^, excluding the Glenbrook Steel Mill emissions as discussed in §2.1 (figure 4). Industrial CO and CO_2_ had estimated uncertainties of 25% giving an *R*_CO_ uncertainty of 50%. These inventory values are consistent with those determined in a similar study in Indianapolis, which calculated *R*_CO_ for residential, commercial, industrial and airport emissions collectively to be 2 ppb ppm^−1^ (excluding emissions from a coal-fired power plant in the centre of the city) [31]. By combining our new observed traffic *R*_CO_ with the inventory CO_2_ emissions, we calculated the revised traffic CO for the Auckland inventory (original traffic CO thought to be overestimated). The inventory traffic CO_2_ and revised traffic CO were then added to the inventory values for the domestic and industrial sectors to calculate a new inventory-based *R*_CO_ for Auckland for the sample collection period (weekday 8.00–18.00). The new weekday daytime inventory *R*_CO_ for Auckland was calculated to be 12 ppb ppm^−1^ (figure 4), which will be used to compare the inventory with the flask observations. Extension of the revised traffic *R*_CO_ to the complete inventory (includes weekday and weekend emissions) while still excluding the emissions from Glenbrook Steel Mill and any biogenic CO_2_ (from wood burning) gives Auckland's *R*_CO_ as 20 ppb ppm^−1^.

The urban sites had an *R*_CO_ of 11 ± 1 ppb ppm^−1^ and had a high correlation (*r*^2^ = 0.8) (figure 3 and electronic supplementary material, figure S.33, table 2). COxs and CO_2_ff had maximum values of 156 ppb and 13 ppm. The urban *R*_CO_ was significantly lower than that of the motorway site (14 ± 1 ppb ppm^−1^), which suggests that Auckland's urban sites observe a strong non-traffic CO_2_ff source (electronic supplementary material, figures S.20–S.27). Based on the *R*_CO_ values for each sector we determined above, we estimate that during the daytime on weekdays, 70% ± 20% of the CO_2_ff emissions observed at the urban sites is from traffic and the remaining 30% is from other sources.

The suburban sites had an *R*_CO_ of 14 ± 1 ppb ppm^−1^ and an *r*^2^ of 0.6 (figure 3 and electronic supplementary material, figure S.31, table 2). COxs and CO_2_ff for the suburban sites had maximum values of 120 ppb and 9 ppm. Relative to the urban sites, the suburban sites had a significantly higher *R*_CO_ that was consistent with the traffic *R*_CO_ that we observed (14 ± 1 ppb ppm^−1^). This implies that CO_2_ff from suburban sites is dominated by traffic emissions between 8.00 and 18.00 on weekdays, the period of flask collection. The suburban sites were further out from the CBD than the urban sites and were therefore expected to have a lower density of commercial and industrial sources, and primarily observe traffic and residential sources (electronic supplementary material, figures S.7–S.13). Since most residential emissions are less active during daytime hours (home heating, natural gas combustion for cooking, lawn mowers, barbecues, etc.) [40], this is consistent with the inventory.

The downwind sites had an *R*_CO_ of 11 ± 1 ppb ppm^−1^ and an *r*^2^ of 0.8 (figure 3 and electronic supplementary material, figure S.32, table 2), very similar to the urban *R*_CO_. COxs and CO_2_ff had maximum values of 138 ppb and 14 ppm. Since the downwind sites were in more elevated locations further from sources, they were expected to be less influenced by local emission sources and would consequently observe more mixed air from a larger region of the city (electronic supplementary material, figures S.14–S.19). For this reason, the emission ratio of the downwind sites was thought to best represent the emission ratio of Auckland as a whole. This observed whole city *R*_CO_ is consistent with the inventory calculated value (adjusted for our observed traffic *R*_CO_) of 12 ppb ppm^−1^. These observations indicate that 70% ± 20% of CO_2_ff in Auckland is from traffic, at least during weekday daylight hours, and is consistent with the inventory estimate of CO_2_ff source sector contributions. This conclusion was limited to the sample collection period. To evaluate the complete inventory, flask collection would be required outside of this sample collection period.

As noted earlier, our traffic *R*_CO_ could be biased low for suburban and urban areas since it is based on motorway observations. Since the traffic *R*_CO_ for the motorway site was based on the assumption that traffic emissions dominated observed emissions, the motorway *R*_CO_ could also be biased low if non-traffic CO_2_ff was observed at the motorway site. Both would result in an overestimate of the traffic contribution of CO_2_ff at the urban, suburban and downwind sites. The consistency between our estimated traffic contribution to CO_2_ff at each site type and the inventory CO_2_ff for Auckland suggest that such a bias in traffic *R*_CO_ is small.

#### (iii) Industrial sites

The industrial sites had the highest *R*_CO_ of all site types of 16 ± 3 ppb ppm^−1^ and an *r*^2^ value of 0.4 (figure 3 and electronic supplementary material, figure S.30, table 2) although with the poorer correlation, they were not significantly different from the motorway site. COxs and CO_2_ff had maximum values of 68 ppb and 5 ppm. Both industrial sites are influenced by a mixture of light industry and traffic, including heavy traffic (electronic supplementary material, figures S.5 and S.6). Excluding Glenbrook Steel Mill emissions, Auckland's inventory industrial *R*_CO_ was calculated to be 1.6 ppb ppm^−1^ [38]. The observed *R*_CO_ is consistent (within uncertainty) with traffic dominating the emissions in these areas. However, the *r*^2^ value of 0.4 is significantly smaller than the other sites, which suggests that temporally varying industrial emission sources could result in variable observed *R*_CO_. For example, certain manufacturing processes only operate intermittently throughout the day, which would contribute to a greater spread of values. Typically, industrial sources have smaller *R*_CO_ due to stricter emission regulations but light industry can strongly vary when not regulated [53,69].

#### (iv) Forest sites

The two forest sites (Titirangi Woodfern Crescent Park and Waitarua Community Centre) were located close to and in the Waitākere Ranges, a regional park that spans over 16 000 hectares (figure 1; electronic supplementary material, figures S.3 and S.4). These sites show little correlation between COxs and CO_2_ff (*r*^2^ = 0) and a small range of COxs and CO_2_ff (−3 to 37 ppb and −1 to 3 ppm), indicating that there were very few local emission sources observed by these sites resulting in signals that were too small to be meaningful.

CO is also produced by oxidation of volatile organic compounds (VOCs) supplied naturally from plants [70–72]. VOC production varies between different types of forests with deciduous trees, particularly eucalyptus, being particularly high VOC producers [73,74]. Limited information is available on VOC and CO production from New Zealand native forests but trees like the pōhutukawa and rātā are in the same family as the eucalyptus and are present in the Waitākere Ranges, so might be expected to produce a higher level of VOCs and CO. While CO derived from VOCs has been shown to be significant in other urban locations [72,75], the Auckland forest sites maintained a relatively low COxs throughout all seasons of the year in our observations. These results implied that VOC-produced CO in the Waitākere Ranges is not a significant contributor and that the CO bias from VOCs is less important for the New Zealand environment.

## 4. Conclusion

*R*_CO_ for traffic in Auckland was determined from the motorway site to be 14 ± 1 ppb ppm^−1^ under the assumption that few non-traffic sources were observed at the motorway site. This was comparable with car fleets seen in locations with similar vehicle fleets and emission controls. The suburban sites had an *R*_CO_ of 14 ± 1 ppb ppm^−1^ that is consistent with a traffic CO_2_ff source and indicates that during the flask collection period (8.00–18.00), Auckland suburban emissions are dominated by traffic emissions. The industrial sites (16 ± 3 ppb ppm^−1^) also had a much greater spread of values, which suggests that a greater mix of sources were present at the industrial sites than the suburban sites and that emissions tend to vary more day to day. The forest sites showed very little correlation due to minimal CO and CO_2_ff sources in the Waitākere ranges but demonstrate that CO production from biogenic VOCs is not a substantial source in the New Zealand environment. The urban and downwind sites had very similar *R*_CO_ of 11 ± 1 ppb ppm^−1^ that were significantly smaller than the traffic *R*_CO_ which suggests that about 70% ± 20% of Auckland's CO_2_ff emissions are from traffic and the remainder are from other sources. Our observations indicate that CO from traffic is overestimated in the Auckland inventory. When we adjust traffic CO emissions to match our observations, we find that our whole-city (downwind) observations are consistent with the overall inventory estimate of *R*_CO_ of 12 ppb ppm^−1^.

Our results demonstrate that observations of CO_2_ff and CO made at the local scale can be used to partition CO_2_ff emission sources within an urban area and improve inventory estimates of emissions.

## Data Availability

Raw flask data and R script used for data analysis/plotting are viewable using the following link. Processed data are in the electronic supplementary material [76]. Analysis was conducted using R v. 4.2.0. Data are available from the Dryad Digital Repository: https://doi.org/10.5061/dryad.1g1jwsv1w [77].
